# Abdominal Supracervical Hysterectomy With Bilateral Salpingo-Oophorectomy as the Surgical Approach for a 22-Week Uterus With Incidental Endometrial Polyp Focal Atypia

**DOI:** 10.7759/cureus.10344

**Published:** 2020-09-09

**Authors:** Maria G Herrera Rodriguez, Divy Mehra, Satesh Saroop, Apurva Srivastav

**Affiliations:** 1 Surgery, Nova Southeastern University School of Osteopathic Medicine, Fort Lauderdale, USA; 2 Ophthalmology, Nova Southeastern University School of Osteopathic Medicine, Fort Lauderdale, USA; 3 Internal Medicine, Nova Southeastern University School of Osteopathic Medicine, Fort Lauderdale, USA; 4 Physical Medicine and Rehabilitation, Nova Southeastern University School of Osteopathic Medicine, Fort Lauderdale, USA

**Keywords:** abdominal hysterectomy, endometrial polyp, focal atypia, fibroid

## Abstract

A 49-year-old perimenopausal female presented with abnormal uterine bleeding (AUB) and chronic lower abdominal pain with associated urinary urgency. The patient elected to have an abdominal supracervical hysterectomy with bilateral salpingo-oophorectomy for a large, symptomatic fibroid uterus. Preoperative ultrasounds revealed a uterine size of 22 x 20 x 17 cm and a 15.9 x 13 x 9 x 9.2 cm subserosal fibroid occupying the majority of the fundus and body of the uterus. Under general anesthesia, abdominal supracervical hysterectomy and bilateral salpingo-oophorectomy with a midline vertical incision were completed. Pathology reported a uterus with multiple leiomyomata as well as endometrial polyps with focal atypical endometrial hyperplasia and squamous metaplasia. Overall, the uterine corpus with one attached adnexa weighed 3433 g and was 25.8 x 20.3 x 15cm. Choice of surgical approach in a hysterectomy depends upon clinical circumstances, the surgeon's technical expertise, and patient preference. Although minimally invasive hysterectomies via vaginal and laparoscopic approaches are now preferred due to decreased hospitalization stays and postoperative recovering times, individualized treatment plans for patients should be considered depending on uterine size and the possibility of not achieving adequate exposure, which may lead to complications. As this case presents, an abdominal hysterectomy is an important option for certain patients where the use of other approaches could pose significant risk.

## Introduction

Abdominal hysterectomy, either total (uterus including the cervix) or subtotal (supracervical), refers to the removal of the uterus via a laparotomy. It is one of the most frequently performed surgical procedures in the United States usually indicated for one of the following broad (often overlapping) categories: uterine leiomyomas, abnormal uterine bleeding (AUB), pelvic organ prolapse, pelvic pain or infection (eg, endometriosis, pelvic inflammatory diseases), and malignant and premalignant disease [1]. Both endometrial polyps and uterine leiomyomas are benign structural abnormalities of the uterus that can present with AUB. Endometrial polyps are localized hyperplastic overgrowths of endometrial glands and stroma around a vascular core that form a sessile or pedunculated projection from the surface of the endometrium [2]. Uterine leiomyomas are noncancerous monoclonal tumors arising from the smooth muscle cells and fibroblasts of the myometrium that are described based on their location according to the International Federation of Gynecology and Obstetrics (FIGO) classification system (Intramural, Submucosal, Subserosal, Cervical) [3,4].

Fibroids are most commonly associated with symptoms such as heavy or prolonged menstrual bleeding, bulk symptoms, reproductive dysfunction, and pain. For those patients that desire fertility, the treatment is myomectomy with an approach depending on location, size, and the number of fibroids (hysteroscopic, laparoscopic, abdominal). For those not desiring fertility, fibroid treatments are aimed at decreasing symptoms and include estrogen-progestin contraceptives, progestin-releasing intrauterine devices (IUDs), gonadotropin-releasing hormone (GnRH) analogs, GnRH antagonists, GnRH agonists, uterine artery embolization, focused ultrasound surgery, and endometrial ablation. If the above therapies are not sufficient and/or patients desire surgical treatment, options include myomectomy and hysterectomy [3].

Endometrial polyps can present with AUB or rarely prolapse. However, many are asymptomatic and are discovered as the result of an evaluation for infertility, a finding of endometrial cells on cervical cytology, or as an incidental finding on endometrial sampling, pelvic imaging, or hysteroscopy [2]. Single or multiple polyps may occur, ranging in diameter from a few millimeters to several centimeters, expressing both estrogen and progesterone receptors. The prevalence of polyps appears to increase with age and risk factors include tamoxifen use, obesity, postmenopausal hormone therapy, and women with Lynch and Cowden syndromes [2,5]. Treatment, when found in isolation, usually requires polypectomy.

## Case presentation

A 49-year-old perimenopausal female with a past medical history of hypertension, hypothyroidism, multiple fibroids, a body mass index (BMI) of 33.2, and a history of iron deficiency anemia presented with AUB and chronic lower abdominal pain with associated urinary urgency. The patient was referred from her primary care doctor due to a large, symptomatic fibroid uterus, and the patient elected to have an abdominal supracervical hysterectomy with bilateral salpingo-oophorectomy. The patient had been diagnosed with leiomyomas in her twenties but had refused surgery until now to maintain fertility. Preoperative transvaginal and pelvic ultrasounds revealed a uterine size of 22 x 20 x 17 cm and a 15.9 x 13 x 9 x 9.2 cm subserosal fibroid occupying the majority of the fundus and body of the uterus. The endometrial thickness on ultrasound was 1.4 cm but with limited visualization due to the fibroid. The ovaries were not visualized. 

Under general anesthesia, a midline incision was performed from the pubic symphysis to and around the umbilicus. Once the abdominal cavity was entered, traction was performed bilaterally on the rectus muscles and peritoneum, extending the incision superiorly and inferiorly. The uterus was exposed and the bowel and omentum were packed with sterile gauze packing. The uterus was externalized and the left fallopian tube was clamped and cut using a sealing instrument. Cauterization and transection were done at the level of the infundibulopelvic ligament. Then, the left ovarian ligament and the left round ligament were clamped, cauterized, and transected. The broad ligament on the left side was then skeletonized. The uterine vessels were identified, clamped twice, cut with scissors, and tied with suture. A similar maneuver was performed on the right side excluding clamping and transection of the right fallopian tube, which was left attached to the uterus, and adequate hemostasis was noted. At this point, the uterocervical junction was identified. The bladder flap was developed and the bladder separated from the uterocervical junction. Using cauterization, the cervix was amputated from the uterus and the cervical stump was reapproximated with adequate hemostasis. Blood loss was approximated at 50 mL with no intraoperative complications. The patient was discharged on postoperative day three with a routine follow-up one week following surgery.

**Figure 1:**
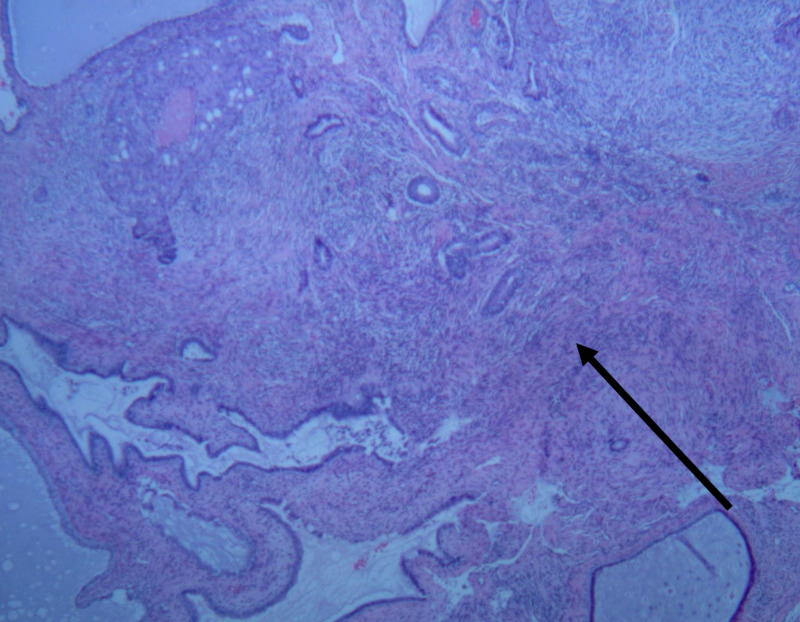
Endometrial polyp

**Figure 2:**
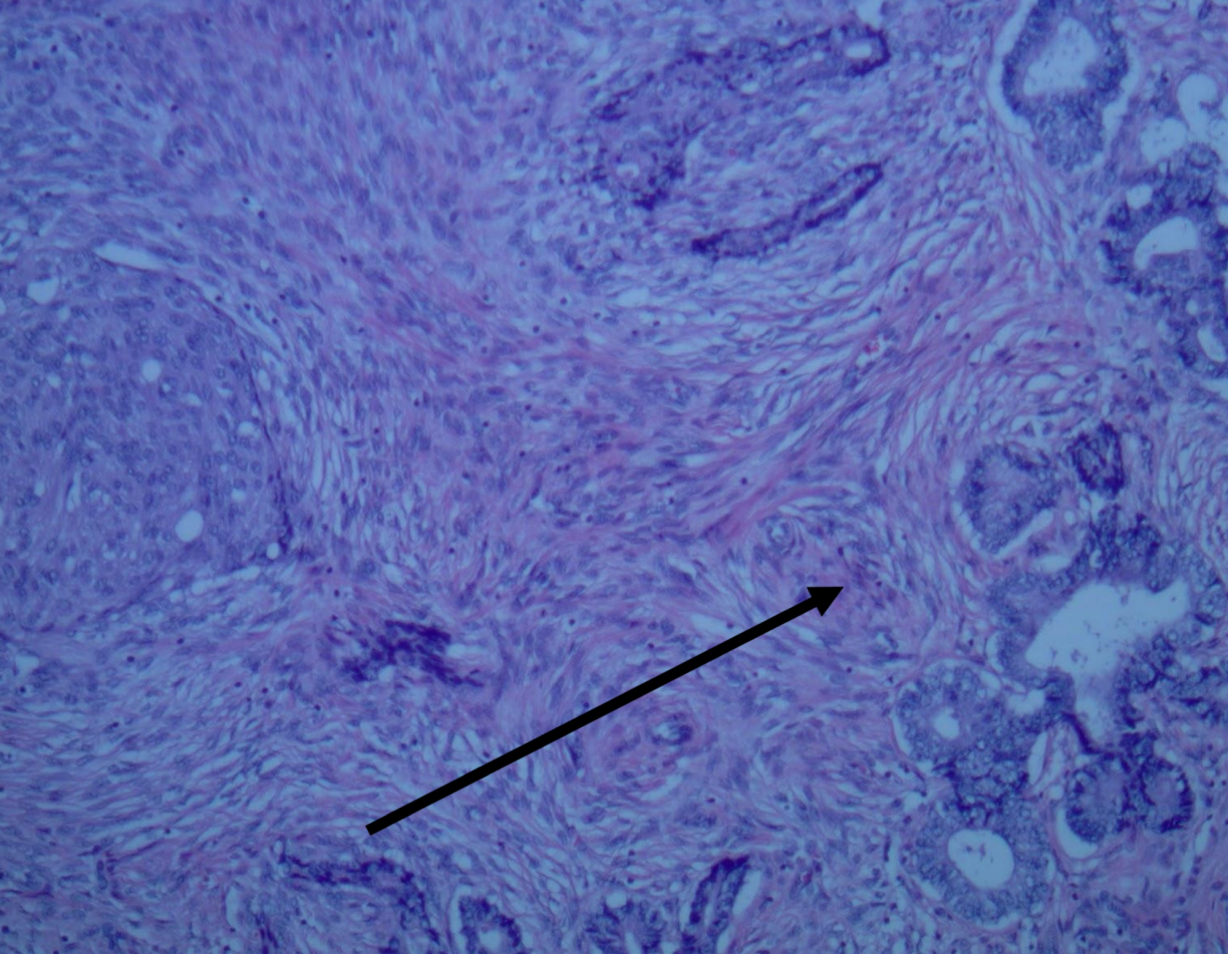
Endometrial polyp with atypical hyperplasia and squamous metaplasia

The uterus and adnexa were submitted for pathology, the results of which were read as a uterus with multiple leiomyomata as well as endometrial polyps with focal atypical endometrial hyperplasia and squamous metaplasia (Figures 1, 2). The myometrial nodules ranged in size from 1.7 x 1.5 x 0.9 cm to 17.3 x 15.2 x 12 cm with mucoid-like and calcified areas of degeneration. The endometrial cavity contained two endometrial polyps, 0.9 cm and 1.4 cm in size, not previously visualized on ultrasound. Overall, the uterine corpus with one attached adnexa weighed 3433 g and was 25.8 x 20.3 x 15cm in size. The endocervix, bilateral fallopian tubes, and ovaries had demonstrated no pathological changes. 

 

## Discussion

The choice of surgical approach in a hysterectomy depends upon clinical circumstances, the surgeon's technical expertise, and patient preference. Although minimally invasive hysterectomies via vaginal and laparoscopic approaches are now preferred due to decreased hospitalization stays and postoperative recovering times, individualized treatment plans for patients should be considered depending on uterine size and the possibility of not achieving adequate exposure, leading to complications. 

According to the American College of Obstetricians and Gynecologists (ACOG), selection of the route of hysterectomy can be influenced by the size and shape of the vagina and uterus, accessibility to the uterus, the need for concurrent procedures, surgeon training and experience, whether the case is emergent or scheduled, and the preference of the informed patient [6]. In terms of size, a measurement approximating to 12 weeks or less usually allows for a vaginal approach, while a size approximating to 18 weeks or above will most likely indicate an abdominal approach. The gray area between 12 and 18 weeks tends to lean towards an abdominal surgical approach as well [7]. Size is a very important consideration for approach due to the amount of exposure achieved to safely remove the uterus with visualization of important structures, such as the vascular pedicles, as well as consideration of uterine shape and mobility. While size is not an absolute contraindication to minimally invasive procedures and there have been cases of large uteruses being removed with these procedures, individualized treatment plans should be made based on expected complications and patient preferences [6,8]. With the uterine size in this case being 22 x 20 x 17 cm and 15.9 x 13 x 9 x 9.2 cm on ultrasound, in addition to a weight of 3433 g, a midline vertical incision abdominal approach was indicated. This was preferred in this case over minimally invasive procedures in order to allow for appropriate visualization and division of important structures, particularly vascular structures and ureters, to minimize complications [9]. Abdominal hysterectomy complications include hemorrhage, infection, thromboembolic disease, ureteral injury, bladder injury, urinary incontinence, bowel injury, bowel obstruction secondary to adhesions, earlier menopause, and vaginal cuff dehiscence [9]. Minimally invasive surgery complications include many of the same possible complications as in open abdominal hysterectomy [9,10].

In order to classically perform a total abdominal hysterectomy, the uterus is grasped at the cornu and pulled up to the incision once the abdomen is entered. The round ligament must be identified and divided. If the ovaries are to be removed, the peritoneal incision is extended from the round ligament to the ovarium hilum, just lateral to the infundibulopelvic ligament. The retroperitoneal space is bluntly dissected with the identification and protection of the ureters bilaterally. The infundibulopelvic ligament is isolated, clamped, cut, and suture-ligated bilaterally. The bladder is mobilized from the anterior surface of the uterus and cervix, clamps are placed on the uterine vessels at the cervicouterine junction, and the vessels are cut and suture-ligated. Then, the cardinal ligaments are divided, the uterus is elevated, and the vagina is clamped. The cervix is amputated from the vagina, sutures are placed at each lateral angle of the vagina, and the remainder of the vagina is sutured closed [9,10].

The large size and weight of the uterine body and fibroid, in this case, did pose a challenge to getting enough exposure to adequately mobilize and safely transect the pedicles. With the proper use of midline incision as well as spending greater than half the total surgical time to adequately expose, identify, and mobilize all structures, we allowed for adequate hemostasis and experienced relative ease removing the uterus and adnexa. 

Endometrial polyps have an overall malignant potential of less than 5% [2,11]. The incidental finding of endometrial polyps with focal atypical endometrial hyperplasia and squamous metaplasia was discussed with the patient at follow-up. At the time of the surgery, the only risk factor for endometrial polyps was obesity, and the only symptom was AUB, likely related to the large fibroids. Because the pathology reported focal atypia with a size less than 1.5 cm and a due to a supracervical hysterectomy being performed, the likelihood of this incidental finding having long-term consequences is low [2,5,11].

## Conclusions

Overall, the current trends and guidelines favor the use of minimally invasive procedures. These procedures have researched benefits and lack true absolute contraindications when choosing a route for a hysterectomy in benign disease. However, as this case presents, the use of abdominal hysterectomies is still an important option for certain patients where the use of other approaches could pose significant risk. Patient preference is also important to consider and patients should be informed of all their options so that they may be active participants in their treatments.

## References

[1] Neis KJ, Zubke W, Fehr M (2016). Hysterectomy for benign uterine disease. Dtsch Arztebl Int.

[2] Nijkang NP, Anderson L, Markham R (2019). Endometrial polyps: pathogenesis, sequelae and treatment. SAGE Open Med.

[3] Laughlin SK, Stewart EA (2011). Uterine leiomyomas: individualizing the approach to a heterogeneous condition. Obstet Gynecol.

[4] Bhatla N, Berek JS, Cuello Fredes M (2019). Revised FIGO staging for carcinoma of the cervix uteri. Int J Gynecol Obstet.

[5] (Facts Views Vis Obgyn). Prevalence and predictors of atypical histology in endometrial polyps removed by hysteroscopy: a secondary analysis from the SICMIG hysteroscopy trial.

[6] (2009). ACOG Committee Opinion No. 444: choosing the route of hysterectomy for benign disease. Obstet Gynecol.

[7] Mohan Y, Chiu VY, Lonky NM (2016). Size matters in planning hysterectomy approach. Womens Health.

[8] Kehde BH, van Herendael BJ, Tas B (2016). Large uterus: what is the limit for a laparoscopic approach?. Autops Case Rep.

[9] Chen B, Ren D-P, Li J-X (2014). Comparison of vaginal and abdominal hysterectomy: a prospective non-randomized trial. Pak J Med Sci.

[10] Einarsson JI, Suzuki Y (2009). Total laparoscopic hysterectomy: 10 steps toward a successful procedure. Rev Obstet Gynecol.

[11] Tabrizi AD, Vahedi A, Esmaily HA (2011). Malignant endometrial polyps: report of two cases and review of literature with emphasize on recent advances. J Res Med Sci.

